# Role of Intracellular Iron in Switching Apoptosis to Ferroptosis to Target Therapy-Resistant Cancer Stem Cells

**DOI:** 10.3390/molecules27093011

**Published:** 2022-05-07

**Authors:** Santhi Latha Pandrangi, Prasanthi Chittineedi, Sphoorthi Shree Chalumuri, Avtar Singh Meena, Juan Alejandro Neira Mosquera, Sungey Naynee Sánchez Llaguno, Ramachandra Reddy Pamuru, Gooty Jaffer Mohiddin, Arifullah Mohammad

**Affiliations:** 1 Onco-Stem Cell Research Laboratory, Department of Biochemistry and Bioinformatics, Institute of Science, GITAM Deemed to be University, Visakhapatnam 530045, India; pchittin@gitam.in (P.C.); sphoortichalumuri98@gmail.com (S.S.C.); 2 CSIR-Centre for Cellular and Molecular Biology (CCMB), Hyderabad 500007, India; avtarjeph@gmail.com; 3 Department of Life Sciences and Agriculture, Armed Forces University-ESPE, Santo Domingo 230101, Ecuador; janeira1@espe.edu.ec (J.A.N.M.); snsanchez@espe.edu.ec (S.N.S.L.); 4 Faculty of Industry and Production Sciences, Quevedo State Technical University, km 11/2 via Santo Domingo, Quevedo 120301, Ecuador; 5 Department of Biochemistry, Yogivemana University, Kadapa 516005, India; reddyprbiotech@yvu.edu.in; 6 Department of Agriculture Science, Faculty of Agro-Based Industry, Universiti Malaysia Kelantan, Jeli 17600, Malaysia

**Keywords:** ferroptosis, apoptosis, cancer stem cells, drug resistance

## Abstract

Iron is a crucial element required for the proper functioning of the body. For instance, hemoglobin is the vital component in the blood that delivers oxygen to various parts of the body. The heme protein present in hemoglobin comprises iron in the form of a ferrous state which regulates oxygen delivery. Excess iron in the body is stored as ferritin and would be utilized under iron-deficient conditions. Surprisingly, cancer cells as well as cancer stem cells have elevated ferritin levels suggesting that iron plays a vital role in protecting these cells. However, apart from the cytoprotective role iron also has the potential to induce cell death via ferroptosis which is a non-apoptotic cell death dependent on iron reserves. Apoptosis a caspase-dependent cell death mechanism is effective on cancer cells however little is known about its impact on cancer stem cell death. This paper focuses on the molecular characteristics of apoptosis and ferroptosis and the importance of switching to ferroptosis to target cancer stem cells death thereby preventing cancer relapse. To the best of our knowledge, this is the first review to demonstrate the importance of intracellular iron in regulating the switching of tumor cells and therapy resistant CSCs from apoptosis to ferroptosis.

## 1. Introduction

**Figure 1:**
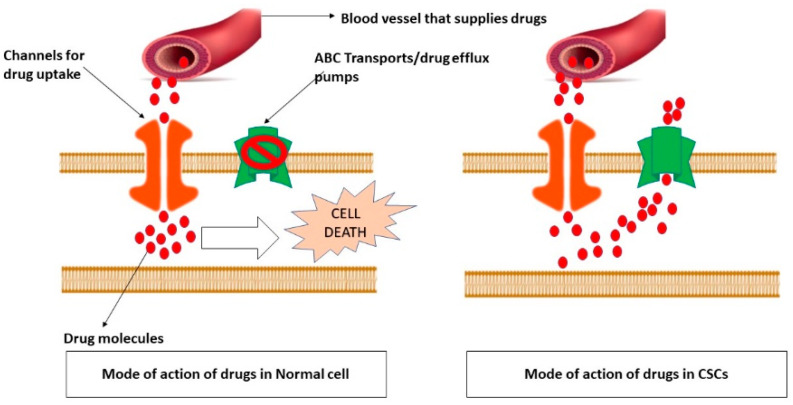
Underlying mechanism of drug resistance in CSCs. To survive in a harsh tumor microenvironment and to withstand the cell death mechanisms CSCs alter the expression of several genes. ABC transporters are one among them which serve as drug efflux pumps. It is believed that upregulation of these drug exports makes CSCs to escape from the effects of anti-neoplastic drugs. This picture describes the mode of action of various drugs in normal cells and CSCs. Usually, under normal conditions, the drug efflux pumps are usually inactivated in the affected cell so that the drug that comes from the circulation would be easily absorbed by that cell with help of certain drug uptake channels. However, in CSCs due to overexpression of ABC transports, although the drug would be absorbed it would be immediately pushed back to the circulation.

Cancer is one of the most alarming health issues worldwide because of its high mortality rate preceding cardiovascular diseases [1]. The most common hallmark of cancer is dysregulated cell death mechanism. It is frequently observed that tumor cells express higher levels of oncogenes that promote cell proliferation; while downregulating tumor-suppressor genes that are involved in regulating cell death events. Therefore, targeting these genes to induce cancer cell death has been widely practiced as a therapeutic strategy for cancer treatment. Although advanced anticancer treatments have made considerable progress, cancer recurrence and drug resistance made these tumors recalcitrant. Figure 1 describes the underlying mechanism of drug resistance in CSCs.

**Figure 2:**
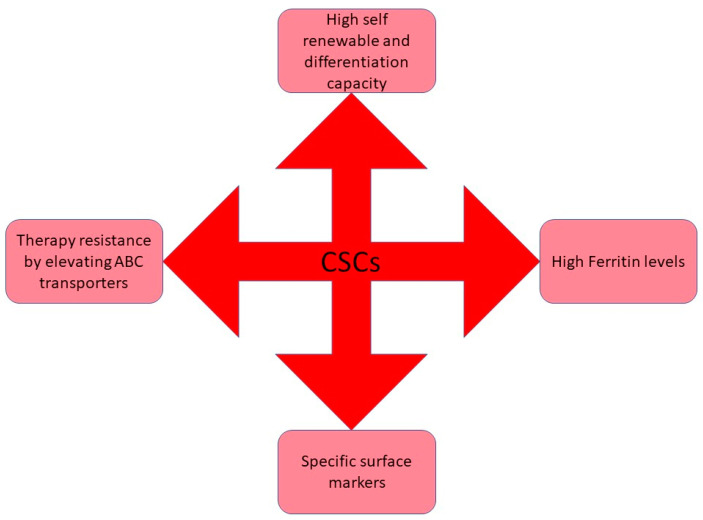
Schematic representation of various characteristic features of cancer stem cells. To survive in the harsh tumor microenvironment CSCs acquire several modifications such as therapy-resistance to abscond the adverse effects of the cancer regimens, self-renewable potential to undergo continuous proliferation, and so on.

For many years, it has been established that human tumors are made up of a diverse population of cells. Cancer Stem Cells are a heterogeneous “swarm” of cells with hierarchical connections comparable to cells in normal tissues, with stem cells functioning as a self-renewing subgroup [2]. Because this group of cells is thought to be capable of beginning new tumor sites, it is also known as “tumor-initiating cells”. They have been examined in the same way as conventional stem cells are, by transplanting them into irradiated, frequently immuno-compromised mouse hosts [3]. The capacity of a subpopulation of cells to serially engraft an animal is an experimental definition of self-renewal and the formation of cell populations with features similar to the remaining tumor cells as proof of differentiation. Bonnet and Dick observed a subpopulation of cells in acute myeloid leukemia patients for the first time in 1997 with similar features of normal stem cells having the properties of self-renewability, differentiation potential and named these cells as Cancer Stem Cells (CSCs), which can initiate tumor [4]. CSCs are defined as the tumor cells, that comprise a diverse population of neoplastic cells that are phenotypically and functionally distinct, correlated with cancer development, metastasis, and resistance to therapy. When these subpopulations of cells were inoculated into an immune-deficient mouse, the mice were diagnosed with cancer demonstrated that CSCs have the tumor-initiating capacity, which may be the reason for cancer recurrence [2]. CSC’s can be distinguished from normal subpopulations based on the expression levels of specific cell surface antigens, most of which are adhesion molecules. The two vital aspects of CSCs are recurrence and resistance to chemotherapy and radiotherapy and this is due to their self-renewability and differentiation capacity [5]. Also, CSCs show high vulnerability towards drug resistance; this might be because these cells remain occult and undetectable during tumor progression or after therapy [6]. Another reason for cancer resistance towards therapy could be the mechanism of ATP-Binding cassettes (ABC) transporters which efflux drugs [7]. Figure 2 schematically represents various characteristic features of CSCs.

Although there are various therapeutic approaches to yield a better prognosis, therapy resistance is a hindrance to overcoming the mortality rate which is accompanied by poor survival outcomes. There are various reasons for a cancer cell to acquire therapy resistance. It should be noted that the chemo/radio cycles vary from patient-patient and is dependent on various factors such as the age of the patient, response to the radiations, and to the antineoplastic drugs, patients’ co-morbidities. However, during chemotherapy or radiotherapy if the patient feels severe discomfort the patient’s therapy cycles would be discontinued and an alternate therapy would be initiated. This is one reason for drug resistance. Hence, new approaches are to be discovered to overcome this problem.

Cell death is crucial for the body’s functioning and maintaining homeostasis, which helps to keep ailments at bay. Apoptosis and necrosis were once thought to be the two primary categories of cellular death [8]. Based on the mechanisms involved, cellular death can be categorized as non-programmed or programmed death mechanisms. There are two types of programmed cell death (PCD): one is non-lytic and the other is lytic cell death. Non-lytic cell death is a kind of apoptosis that generates apoptotic bodies that are retrieved by phagocytes and do not require an inflammatory response [9]. Necroptosis and pyroptosis are two lytic types of cell death. These types of cell death induce intracellular components to seep out, triggering a substantial inflammatory response and inflammatory death. Necrosis is a term used to describe the process of irreparable cell damage and eventual cellular death produced by chemical or physical stimulation under harsh settings [10]. The disintegration of cellular membranes, edema of cells and cytoplasmic vesicles, and the breakout of cellular contents are the most prominent features of necrosis; however, during necrosis chromatin does not agglutinate [2].

On the contrary, in 2012, Stockwell hypothesized a novel kind of cellular death known as ferroptosis. Ferroptosis is a kind of cell death mechanism triggered by peroxidation of lipids in which iron is involved. Iron is a vital inorganic element for various biological processes. For example, hemoglobin is an essential pigment of Red Blood Cells that plays a major role in carrying oxygen to various cells. To our knowledge, we know that hemoglobin is an iron-containing globular protein suggesting that iron is required to the body to carry oxygen. Apart from oxygen transport, iron also plays a crucial role as a co-factor for enzymes that are involved in DNA synthesis, cell cycle events, as well as in detoxification process. Since it is directly intertwined with cell proliferation and growth, it is likely that iron metabolism would be altered in tumor cells, which have substantial growth. Studies on tumor cell iron metabolism revealed that tumor cells required greater iron concentrations and that iron absorption protein genes were considerably over-expressed. Literature studies related to iron metabolism and cancer suggested that iron is one of the major pre-requisites for cancer cells and cancer stem cells. Although CSCs require bulk iron than compared to cancer cells. Changes in mitochondrial atrophy, mitochondrial phenotype, and a increase in membrane thickness are other important characteristic features of ferroptosis. Various kinds of cell death have been found to significantly influence the progression of a wide range of chronic disorders. This review focuses on apoptotic and ferroptotic mechanism and their role in inducing cell death in drug resistant CSCs.

## 2. Identification of CSCs

The self-renewability and infinite progression property of the CSCs drive cancer cell proliferation, drug resistance, metastasis, and relapse [11].These cells could act as therapeutic targets and could be detected by the presence of stemness marker expression such as CD133, CD44, CD24,etc., and pluripotency factors such as Sox2, Oct4, and Nanog and by their capacity to form spheroids [12,13].

However, it should be noted that although CD 133 expressing CSCs display stem-cell-like properties using the same for identification and isolation of CSCs has become controversial because of their expression in the glandular epithelium is few tissues making it difficult to differentiate between CSCs and non-stem like tumor cells [14,15]. Secondly, when the original tumor morphology was xeno transplanted the CD 133^+ve^ subpopulations could not reciprocate the same as seen in control. On the contrary, the CD 133^-ve^ subpopulation showed effective recapitulation towards the original morphology of the tumor suggesting that CD 133 might not be a unique marker in identifying the CSCs population [16].

On the other hand, CD 44 is a transmembrane glycoprotein that is upregulated in various malignancies characterized by expressing alternatively spliced variants. In humans, this gene is encoded by 10 standard constant exons (CD 44s) and 9 variant exons that play a major role in distinguishing various isoforms coupled with the 10 constant exons (CD 44v) [17]. Research is more focused towards investigating the importance of CD 44 isoforms in the pathophysiology of CSCs. Interestingly, Brown et al., observed a switch of CD 44v to CD 44s during EMT induction suggesting the importance of isoform switching in tumor metastasis [18]. Several studies indicated the role of CD44 isoform in tumor invasion and metastasis with poor survival outcome [19,20].

Additionally, ALDH1, an isoform of Aldehyde Dehydrogenase which mediates the detoxification of toxic aldehyde intermediates produced due to particular anticancer agents, was recently reported to confer resistance in CSCs due to therapy [21]. It is demonstrated that ALDH expression regulates cell cycle checkpoints and DNA repair pathways in cancer, thereby promoting platinum resistance in ovarian cancer [22].

Apart from these CD26 came into light as one of the surface markers due to their critical role in tumor progression, apoptosis, and immunomodulation. The underlying mechanism of metastasis’ promotion by CD26 is because of its aptitude to interact with the extracellular matrix proteins particularly type-I and II fibronectins and collagens [23]. It has been observed that HT-29, HRT-18, T84, SW-620, and SW-480 cell lines showed a drastic increase in expression of CD26 when exposed to potent antineoplastic drugs such as 5-Flurouracil, platinum-based drugs [24]. These finding suggest that development of novel antineoplastic drugs that could potently arrest stem cell expression in tumor tissues is necessary. Table 1 represents various stem cell surface markers expressed in different malignancies.

## 3. CSCs Plays an Essential Role in Drug Resistance and Cancer Relapse

Several studies have reported that the cancer patients who have shown a better prognosis during the treatment were again diagnosed with cancer after a certain period. This condition is termed cancer relapse. CSCs are responsible for cancer recurrence along with drug resistance. The reason might be due to the property of these cell store main in the quiescent phase and grow slowly, making these cells escape from the potent anti-cancer drugs [36]. CSCs consume time in the maturation and differentiation, which might be the reason for cancer relapse during treatment. In addition, CSCs resemble the normal stem cells, retaining the properties to survive against therapy, hypoxic condition, metabolic stress, and starvation by enhancing autophagy pathways [37]. CSCs acquire various proteins by enhancing autophagy, and this process contributes to drug resistance [38,39]. For instance, several studies showed high expression of CD133, commonly referred to as prominin-1, is correlated with drug resistance in CSCs. Investigations revealed that platinum and paclitaxel, the first-line drugs used in lung cancer, showed resistance by elevating the expression of ABC transporters mediated by CD133 expression [40]. Surprisingly, ALDH1, an isoform of Aldehyde Dehydrogenase which mediates the detoxification of toxic aldehyde intermediates produced due to particular anticancer agents, was recently reported to confer resistance in CSCs due to therapy [21]. It is demonstrated that ALDH expression regulates cell cycle checkpoints and DNA repair pathways in cancer, thereby promoting platinum resistance in ovarian cancer [22].

## 4. Approaches to Target CSCs Death

CSCs adapt to hypoxia and metabolic stress, which are the hallmarks of the tumor micro-environment, with the help of iron. Thus, this indispensable nutrient maintains all the necessary conditions required to develop a tumor microenvironment [41]. A study showed that iron chelation decreased the expression of surface markers such as CD133, CD44, CD24, which are the CSC markers [42]. In contrast, the expression of these markers was overturned with iron supplementation, demonstrating that iron plays an essential role in maintaining stemness in CSCs [43,44,45]. Therefore, targeting iron metabolism might effectively inhibit CSC growth, preventing cancer recurrence and drug resistance. Conversely, iron is also a key player in regulating ferroptosis, regulated iron-dependent necrosis [46]. The underlying principle through which CSCs abrogate ferroptosis involves different ionic iron forms, such as ferrous iron (Fe^+2^) and ferric iron (Fe^+3^). Among these two forms, ferric iron promotes cancer growth by enhancing ribonucleotide reductase enzyme activity, the critical enzyme for nucleotide biosynthesis [47]. In contrast, ferrous iron activates lipoxygenase, thereby contributing to ferroptosis [48]. Interestingly, cancer and CSCs store bulk of iron in ferric state (ferritin), limiting the availability of ferrous iron, thereby inhibiting ferroptosis [49].

## 5. Characteristic Features and Mechanism of Apoptosis

Apoptosis, or regulated cell death, is characterized by distinctive structural features and energy-dependent molecular ways. Apoptosis is thought to be an indispensable component of several processes, including, immune system development and function, normal cell turnover, embryonic development, and chemical-induced cell death, hormone-dependent atrophy [33,50]. Inappropriate apoptosis seems to have a vital role in a wide range of human disorders, including neurodegenerative diseases, autoimmune disorders, ischemia damage, and many kinds of cancer. The potential to regulate a cell’s life or death is acknowledged for its enormous therapeutic potential, decreases in cellular size, damage of connections, and detachment from surrounding cells are the major morphological characteristics of apoptosis [51]. Apoptosis is defined morphologically by cell size decreases, loss of connections, and separation from neighboring cells. The cytoplasmic density upsurges slowly, and the mitochondrial membrane potential diminishes. The nucleoplasm is concentrated in the nucleus during apoptosis, the nucleolus is disrupted, and DNA is fragmented into 180–200 bp pieces [52]. The whole cell retains its cytosolic structure, with vesicle generation that eventually splits and wraps the apoptotic cell into numerous apoptotic vesicles. This procedure does not entail the discharge of cellular contents, nor does it stimulate the inflammatory response. Cancer cells abscond the apoptotic pathway by modulating certain transcription factors such as STAT-3, NF-κB, antiapoptotic and pro-apoptotic proteins [53].

Intracellular cysteine proteases formerly known as caspases are responsible for the molecular machinery apoptosis. The name of caspases has been derived from its mode of action towards their substrates by cleaving their target proteins at aspartic acid residues. Hence it could be abbreviated as cysteine aspartic acid-specific proteases. Since apoptosis is tightly regulated and activated under stress conditions these enzymes are present as inactivating zymogens which are activated under the specific stimulus. Upon activation, zymogens are cleaved into major and minor subunits thereby generating an N-terminal prodomain. The active enzymes are hetero tetramers made up of two larger and two smaller subunits, each with two active sites each.

Depending on the type of stimuli apoptosis could be classified into intrinsic and extrinsic pathways. Members of the Tumor Necrosis Factor family of cytokine receptors, such as Fas and TNFR1, can activate the extrinsic pathway [54,55]. These proteins link adapter proteins to their cytosolic DDs, such as Fadd, which subsequently binds DED-containing pro-caspases, namely pro-caspase-8. The intrinsic pathway could be triggered by the release of cytochrome c from mitochondria, which can be triggered by a variety of stimuli, including increases in the amounts of pore-bearing pro-apoptotic Bcl-2 family members like Bax. Cytochrome c interacts with and activates Apaf-1 in the cytosol, permitting it to bind and stimulate pro-caspase-9. Active caspase-9 (intrinsic) and caspase-8 (extrinsic) have been found to cleave and trigger caspase-3, an effector protease.

## 6. Mechanism of Ferroptosis

**Figure 3:**
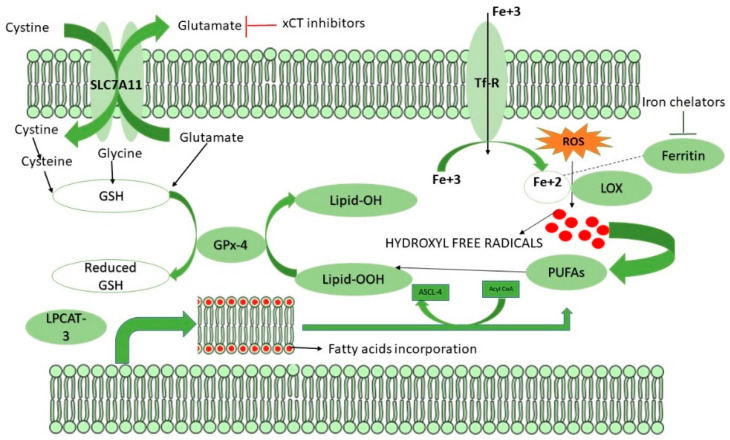
Possible mechanisms of inducing ferroptosis. Ferroptosis is a caspase independent cell death characterized by generation of lipid peroxides. Usually, under normal homeostasis if a cell needs to undergo ferroptosis, the cell enhances the generation of lipid peroxides with the help of various genes. Briefly, under unfavourable conditions, cellular ROS would be elevated which signals the cell membrane to incorporate fatty acids via LPCAT-3 enzyme, the fatty acids incorporated cell membrane would be further esterified by the ASCL-4 leaving poly unsaturated fatty acids (PUFAs) and acyl CoA. PUFAs would then interact with the hydroxyl free radicals produced by the generated ROS via LOX enzyme which requires ferrous iron through a process called Fenton reaction. Interestingly, there are two ways to induce ferroptosis in CSCs. The first mechanism to induce ferroptosis is by inhibiting SLC7A11. SLC7A11 is a cys-glu antiporter that imports cystine inside the cell by simultaneously exporting glutamate in a 1:1 ratio. The imported cystine is then reduced to two molecules of cysteine, which is then coupled with glutamate and glycine to form GSH. The formed GSH now acts as a co-factor for GPX4 and catalyses the conversion of lipid peroxides into lipid hydroxides, thereby halting ferroptosis. Secondly, ferroptosis could also be induced by iron chelators which degraded ferritin thereby enhancing LOX activity.

Polyunsaturated fatty acids (PUFA) are exclusively present in phospholipids which are more susceptible to lipid peroxidation and are the major drivers for ferroptosis [56]. Ferroptosis, a non-apoptotic cell death characterized by aberrant lipid oxidation, ROS accumulation, elevated ferritin, transferrin, and GSH levels [57]. Three crucial enzymes regulate ferroptosis, including Acyl CoA synthetase long-chain family member-4 (ACSL-4), Lysophosphotitdyl Choline Acyl transferase-3 (LPCAT3), and Lipoxygenases (LOX) [58]. In the early stage, LPCAT-3 catalyzes the incorporation of arachidonic and adrenic acids into the lipid bilayer, which acts as the substrate for lipid peroxidation [59]. ACSL-4 then catalyzes the esterification of the incorporated arachidonic and adrenic acids with coenzyme-A that results in the formation of Acyl CoA, generating a lipid target pool for peroxidation [60]. The generated Acyl CoA undergoes β-oxidation or anabolic PUFA biosynthesis. As the biosynthesis of PUFA is completed, these undergo lipid peroxidation in the presence of free radicals generated via the Fenton pathway, which is catalyzed by the enzyme LOX in the presence of ferrous iron [61]. Figure 2 represents the mechanism of ferroptosis and how CSCs overcome the process. But cancer and CSCs protect themselves from ferroptosis by limiting the availability of divalent iron through rapid oxidation of ferrous iron to ferric iron, which enhances nucleotide biosynthesis, thereby promoting cell proliferation [62]. Another approach by which CSCs escape ferroptosis is synthesizing anti-oxidant glutathione (GSH) [48], which serves as an electron donor for Glutathione peroxidase 4 (GPX4) to protect cancer cells from ferroptosis by peroxidation of generated lipid ROS to reduced lipid alcohols [63]. Figure 3 represents the mechanism of ferroptosis as well as its induction in drug resistant CSCs

## 7. Switching Apoptosis to Ferroptosis in Drug-Resistant CSCs

CSCs, like healthy tissue stem cells, are considered to be resistant to apoptosis in order to proliferate. Although this has not been extensively studied, it is suspected that this is triggered by intracellular pathways that lead to barricades in apoptosis. Scientific proof for this in compacted tumors has come primarily from brain tumors, such as glioblastoma (GB) [64]. Resistance to an apoptotic cell death was demonstrated in CD133+ GBSCs isolated from patients with glioblastoma compared to CD133, non-CSC fractions by raised confrontation to various chemotherapeutical agents, which was related with increased expression of various anti-apoptotic mRNAs, including BCL-XL, BCL-2, IAPs, and FLIP [28]. Scientific proof for resistance to apoptosis in GBSC has been established by demonstrating that TRAIL tolerance in CD133+ GBSCs in contrast with CD133 cells is associated with methylation in the promoter region of the CASP8 gene because of the suppression of caspase 8 gene expression. Although the DNA demethylation molecule 5-Aza-20-deoxycytidine could rescue caspase-8 gene expression, it was insufficient to make the GBSC susceptible to TRAIL. There were no alterations in cFLIP expression between CD133+ and CD133 cells, ruling out a function for this protein in resistance [29].

On the other hand, studies showed ferroptosis which is a non-apoptotic cell death dependent on iron reserves sensitizes CSCs to undergo cell death. Cancer cells can be differentiated from normal cells due to altered lipid metabolism, ROS accumulation, and high ferritin levels [65]. Ferritin is an iron reservoir that stores excess iron in a ferric state, is absorbed from the intestinal epithelial cells, and exported by ferroportin [66]. A significant number of genes that regulate the import and export of iron to various cells and tissues are dysregulated in cancer and CSCs. Studies showed that CSCs exhibit elevated ferritin levels inside the cell compared to regular counterparts [62]. Hence, targeting the ferritin levels in cancer cells and CSCs by ferroptosis pathway may bear a therapeutic value and sensitize to anticancer agents [49].But these cells protect themselves from cell death due to ROS accumulation by increasing glutathione levels, a lipid peroxide scavenging molecule via., up-regulating SLC7A11 gene [67]. On the other hand, a recent study proved that ferroptosis inducers such as erastin target CSCs in a tumor and induce CSC’s death, thereby preventing cancer recurrence and overcoming drugs resistance [68].

## 8. Role of GSH in Ferroptosis

In mammalian cells, GSH, a tripeptide comprising glutamate, cysteine, and glycine in equal ratios, plays a vital role in maintaining intracellular redox homeostasis by scavenging the generated peroxides. Although glycine and glutamate are present inside the cell, cysteine is imported into the cells through the SLC7A11 gene, formerly called xCT-System [69]. The enzyme glutathione peroxidase-4(GPX4) utilizes GSH as co-factor and develops resistance to ferroptosis by eliminating the lipid peroxides [70]. There are many isoforms of GPX which play a dual role in CSCs proliferation, drug resistance, metastasis, and recurrence. For instance, GPX1, dramatically downregulated in various cancers, is a negative regulator for cancer progression. Interestingly, GPX1is over expressed, diminishing the clonogenicity propensity and leading to cell death [71]. However, low GPX1 levels contributed to metastasis and chemoresistance in CSCs [72]. In contrast, GPX-4 inhibition resulted in the CSCs being more prone to ferroptosis [73]. Thus, cancer cells and CSCs need a bulk amount of GSH to catalyze the reactions mediated by GPX-4. Since these cells need efficient GPX-4 enzyme activity, they might enhance GSH synthesis by up-regulating the SLC7A11 gene, a cysteine/glutamate antiporter. Thus, antiporter exports intracellular glutamate and simultaneously imports one cysteine molecule [67].

## 9. Targeting CSCs via Ferroptosis

When compared with normal cancer cells, CSCs are more sensitive towards ferroptosis. Studies showed that the CD44 variant (CD44v), a cell adhesion molecule widely expressed in various CSCs controls the intracellular levels of reduced GSH by directly interacting with the h SLC7A11, thereby promoting tumor growth [40]. In the transgenic mouse model of gastric cancer, when CD44v was knocked out, the tumor growth was suppressed, and there was a significant loss of the SLC7A11 gene. Additionally, it resulted from the inactivation of p38 and p21, thereby inducing cell death [19]. Salinomycin, ironomycin, and ebselen are ferroptosis-stimulating agents that displayed promising outcomes in targeting CSCs, particularly in breast cancer. A notable synergistic effect was observed when the ferroptosis activators were used along with chemotherapeutic drugs [74]. Surprisingly, iron crucial for regulating ferroptosis acts as a double-edged sword in CSCs growth. A significant body of evidence suggested elevated ferritin levels, an iron storage protein, and ferroportin, an iron exporter protein in various cancers and CSCs [44,68]. Interestingly the elevated level of ferritin is mediated by the transferrin receptor (TfR), which regulates the entry of iron that is specifically bound to transferrin (Tf)through receptor-mediated endocytosis [75]. Elevated TfR levels in CSCs demonstrated high potential to form spheroids, and uptake of labile iron into the cell as compared to cancer cells. Increased iron reserve in the form of ferritin promotes spheroid forms of CSCs which are more susceptible to ferroptosis. [57]. Although CSCs are sensitive to ferroptosis, CSCs develop resistance by elevating GSH, an antioxidant that plays a protective role via inhibiting ferroptosis. Therefore, targeting GSH metabolism might also effectively induce ferroptosis in CSCs [76].Evidence from increased number of various studies show that GSH plays an essential role in self-renewal and chemoresistance in pancreatic CSCs. An exponential decrease in CSC survival, proliferation rate, and self-renewability capacity was observed when the GSH content was depleted. Interestingly, depletion of GSH in CSCs responded to the gemcitabine, a commercially available drug used to treat various cancers, suggesting that elevated level GSH in CSCs contribute to chemoresistance [77].

On the other hand, downregulation of SLC7A11 expression, usually associated with GSH metabolism, can also be the therapeutic strategy to target CSCs. Since SLC7A11 expression correlates with GSH levels inside the cell, it is not astonishing that SLC7A11 over expression enders the cells resistant to various drugs but also associated with enhanced spheroid formation [26]. In Colorectal cancers, when the level of the SLC7A11 gene is silenced, it reduces stemness and sensitizes CSCs to ferroptosis [78]. Furthermore, various other studies have demonstrated that targeting SLC7A11 by siRNA or inhibitors sensitizes drug-resistant CSCs. For instance, suppression of the SLC7A11 gene results in re-sensitization of CSCs when treated with cold plasma, method for cancer treatment that works by elevating the intracellular ROS production [79]. While, the role of SLC7A11 in therapeutic resistance is still unclear, it could be demonstrated by numerous studies showing that SLC7A11, a cystine/glutamate antiporter, imported specific anti-cancer molecules and drugs. Interestingly, the downregulation of this gene resulted in multidrug resistance in MCF-7 breast cancer cell lines [70]. Hence, more studies are warranted to understand and decipher the involvement of SLC7A11in the regulation of ferroptosis and drug resistance. Table 2 summarizes various drugs and their mechanism of action to induce ferroptosis in therapy-resistant CSCs.

## 10. Conclusions

Cancer Stem Cells (CSCs) are derived from mutated adult stem cells; serve as a model to study tumorigenesis, tumor growth, and metastasis, and are responsible for the aggressiveness of malignant tumors [92,93]. The existence of CSCs could explain why current treatments for various malignancies are not able to eradicate tumor cells and have reached a “therapeutic plateau” because these therapies target the bulk of cancer cells leaving behind therapeutic resistant CSCs. CSCs undergo self-renewal, recapitulate the phenotype of the tumor from which they are derived, develop into phenotypically diverse cancer cell populations, proliferate extensively, and are responsible for the development of resistance against chemo/radiotherapy, tumor recurrence, and relapse [94]. Ferroptosis, an iron-dependent caspase-independent cell death pathway, was recently reported as a promising mechanism to tackle CSCs among different malignancies as they exhibit an enhanced dependence on iron for growth and hence are dramatically more susceptible to iron depletion than non-CSCs [95]. An approach to understanding tumor relapse and recurrence and resistance to current treatment modalities might lead to the identification of novel treatment targets. The present review is an attempt to through light on the mechanisms of ferroptosis as a potent way to target drug resistance, tumor relapse, and recurrence which might lead to novel cancer interventions and therapeutic approaches.

## Figures and Tables

**Table 1:** Stem cell surface markers in various cancers.

Stem Cell Surface Marker	Cancer Type	References
CD44	Breast, Colon, Head and Neck, Liver, Lung	[5,25,26,27]
CD90	Brain, Liver	[28]
CD133	Breast, Colon, Brain, Liver, Lung, Endometrial	[16,29]
CD271	Head and Neck, Skin	[30,31]
ALDH1	Breast, Endometrial	[5,32,33]
EpCAM	Colon, Liver	[3]
CD24	Breast, Colon	[27]
CD166	Lung, Colon	[34,35]
CD26	Colorectal, colon with lung metastasis, breast, melanoma	[23]

**Table 2:** Summarizes various drugs and their mechanism of action to induce ferroptosis in therapy-resistant CSCs.

Drug	Mechanism	Tumor Type	References
**Iron chelators**	Degrades ferritin, promotes LIP expression, and induces HMOX-1 expression	Breast, ovarian, colorectal, pancreatic, cervical	[80,81,82,83,84]
**SLC7A11 inhibitors**	Inhibits cysteine uptake thereby reducing glutathione levels	Glioma, breast, lung, melanoma, cervical, prostrate, neuroblastoma	[25,78,79]
**GPx-4 inhibitors**	Inhibit the enzyme activity of GPx-4 thereby enhancing lipid peroxides	Leukaemia, lymphoma, sarcoma, ovarian cancer, pancreatic, lung	[85,86,87]
**Cysteinase**	Glutathione inhibitor that degrades cysteine and cystine	Prostrate, pancreatic, chronic lymphocytic leukaemia	[88,89]
**FINO_2_**	Indirectly inactivates GPx-4; promotes ROS accumulation by oxidizing PUFAs and ferrous iron	Fibrosarcoma	[90,91]
